# Augmenting the Efficacy of Immunotoxins and Other Targeted Protein Toxins by Endosomal Escape Enhancers

**DOI:** 10.3390/toxins8070200

**Published:** 2016-07-01

**Authors:** Hendrik Fuchs, Alexander Weng, Roger Gilabert-Oriol

**Affiliations:** 1 Institut für Laboratoriumsmedizin, Klinische Chemie und Pathobiochemie, Charité – Universitätsmedizin Berlin, Campus Virchow-Klinikum, 13353 Berlin, Germany; 2 Institut für Pharmazie, Freie Universität Berlin, 14195 Berlin, Germany; weng@zedat.fu-berlin.de; 3 Department of Experimental Therapeutics, BC Cancer Research Centre, Vancouver, BC V5Z 1L3, Canada; rgilabertoriol@bccrc.ca

**Keywords:** endosomal escape, efficacy enhancers, targeted toxins, immunotoxins, cytosolic drug delivery, controlled drug release, cancer treatment, endocytosis

## Abstract

The toxic moiety of almost all protein-based targeted toxins must enter the cytosol of the target cell to mediate its fatal effect. Although more than 500 targeted toxins have been investigated in the past decades, no antibody-targeted protein toxin has been approved for tumor therapeutic applications by the authorities to date. Missing efficacy can be attributed in many cases to insufficient endosomal escape and therefore subsequent lysosomal degradation of the endocytosed toxins. To overcome this drawback, many strategies have been described to weaken the membrane integrity of endosomes. This comprises the use of lysosomotropic amines, carboxylic ionophores, calcium channel antagonists, various cell-penetrating peptides of viral, bacterial, plant, animal, human and synthetic origin, other organic molecules and light-induced techniques. Although the efficacy of the targeted toxins was typically augmented in cell culture hundred or thousand fold, in exceptional cases more than million fold, the combination of several substances harbors new problems including additional side effects, loss of target specificity, difficulties to determine the therapeutic window and cell type-dependent variations. This review critically scrutinizes the chances and challenges of endosomal escape enhancers and their potential role in future developments.

## 1. Introduction

Targeted protein toxins represent one of the high hopes for future drugs in the fight against cancer. They consist of a targeting domain chemically coupled or recombinantly fused to a toxic payload for tumor cell killing [1]. In most of the cases, the targeting moiety is an antibody-based domain including various forms of full regular antibodies, single chain antibodies, bivalent or bispecific diabodies and minibodies [2,3,4,5], and this type of targeted toxins is therefore called immunotoxins. The diversity of these molecules allows the selection of very specific structures for the targeting of tumor cells, however, the protein toxins take their effect inside the cytosol. Therefore, targeting of the toxin to the cell surface is a prerequisite for specificity but not sufficient for efficacy. In many constellations, the toxin remains ineffective since: (1) the antibody is poorly internalized and remains bound to the outside of the cells; (2) is recycled back to the cell surface after internalization; or (3) transported to the lysosomes where it is degraded [6,7,8]. Although these fundamental issues are known for decades, the problems have not been solved yet and no antibody-targeted protein toxin has been approved for tumor therapeutic applications by the authorities to date. It is obvious that a major problem is the endosomal escape within sufficient time. To overcome this drawback, many strategies have been described including approaches to redirect the toxins to endogenous cellular membrane transport complexes of the biosynthetic pathway in the endoplasmic reticulum, disrupting the endosomes, weaken the membrane integrity of endosomal membranes or diving through these membranes with the assistance of cell penetrating peptides [9,10,11,12].

All strategies including physicochemical techniques require molecules that interact more or less directly with membranes and comprise essentially small chemical molecules, secondary metabolites, peptides and proteins. A common feature of all these substances is that they are per se not target-specific and distribute with other kinetics than the targeted toxins. Therefore, a number of requirements can be formulated for endosomal escape enhancers: (1) they must not be toxic for regular cells; (2) they must not mediate the cytosolic uptake of the targeted toxin into off-target cells; (3) their presence at the site of action must by synchronized with the presence of the targeted toxin; (4) they must be biodegradable or excretable; and (5) they must not substantially interfere with metabolic processes of the organism, e.g., interact with hormones. It is easy to recognize that most of these criteria can only be sufficiently tested in in vivo systems, however, most of the published endosomal escape enhancers have only be tested in vitro, which may explain the lack of success of these strategies in clinical applications. The present review describes the different approaches to augment the efficacy of targeted toxins by endosomal escape enhancers including chemical, viral, bacterial and eukaryotic substances. It then discusses the pros and cons of the different techniques with regard to the mentioned requirements and weighs the prospects and risks for future clinical development of the different strategies.

## 2. Chemical Enhancers

Many different chemical enhancers have been described in the literature to augment the efficacy of targeted toxins. These compounds are generally small chemical molecules with great variability that increase in some way or another the cytotoxicity of targeted toxins. Within the chemical enhancers, the compounds can be classified in several subgroups depending on their chemical nature and molecular mechanism. This includes the subgroups of lysosomotropic amines, carboxylic ionophores, calcium channel antagonists, and other organic compounds. Detailed information is provided for each of the subgroups in the following subsections.

### 2.1. Lysosomotropic Amines

When cells are treated with targeted toxins, they are commonly internalized into cells by receptor-mediated endocytosis and then undergo their intracellular routing. A substantial amount of targeted toxins are not able to reach their intracellular target and accumulate in the lysosomes where they are degraded. This is one of the main limitations of targeted toxins and results in the decrease of their efficacy [13]. Lysosomotropic amines increase the lysosomal pH and therefore interrupt the degradation of proteins by pH-dependent lysosomal enzymes [14]. Since they have their site of action inside the lysosomes, only targeted toxins that mainly accumulate in these organelles are likely to be enhanced. A list of the lysosomotropic amines that have been reported to facilitate the cytosolic delivery of targeted toxins is presented in Table 1.

Ammonium chloride was firstly used to enhance the cytotoxicity of an anti-melanotransferrin-ricin A chain immunotoxin [15]. The cytotoxic effects were enhanced roughly 100-fold and this encouraged the investigation about combinatorial effects of ammonium chloride with further targeted toxins. The most prominent effects were observed in the case of targeted toxins based on ricin toxin A chain (RTA). As an example, the cytotoxicity of three RTA immunotoxins directed to the cell surface antigens Thy 1.2 (cluster of differentiation 90, CD90), melanotransferrin and CD5 was augmented by 5.7, 42 and 6700 folds, respectively [16]. On the contrary, the cytotoxicity of diphtheria toxin was blocked when it was administered to cells in the presence of ammonium chloride [17], which was not surprising since it has been known that inhibition of the vacuolar-type H(+)-ATPase also blocked diphtheria toxin toxicity [18].

Chloroquine is another lysosomotropic amine that has been extensively studied. The highest enhancing effects were again observed in the case of targeted toxins containing RTA. The immunotoxin T101-RTA was enhanced 2500-fold by chloroquine [16]. Chloroquine was also efficient in enhancing the cytotoxic effects of targeted toxins composed of gelonin [19], pokeweed antiviral protein [20] and saporin [21], although the enhancing effects were moderate.

Other lysosomotropic amines that are able to enhance the efficacy are amantadine [22], methylamine, dimethylamine, trimethylamine [16], lipopolyamines [23], quinacrine (mepacrine) [19] and glycylphenylalanine 2-naphthylamide [24]. All compounds were simultaneously co-administered with the targeted toxins and are believed to act in the same way as proton reservoirs through their amine groups thus preventing the acidification of the lysosomes.

### 2.2. Carboxylic Ionophores

Carboxylic ionophores have been successful in enhancing the delivery of targeted toxins to the cytosol. Carboxylic ionophores integrate in lipid bilayers and mediate the exchange of monovalent cations across the membranes. An example of this mechanism is the exchange of sodium ions and protons by monensin [35]. When administered to cells, carboxylic ionophores increase the lysosomal pH and this finally results in blocking protein degradation inside the lysosomes [36]. At the same time, the inhibition of lysosomal degradation may augment the cytotoxic effects of targeted toxins as observed for lysosomotropic amines. A list of such carboxylic ionophores is available in Table 2.

Monensin is the most well studied compound within the carboxylic ionophores. The highest enhancement effects were observed in the case of targeted toxins comprising ricin A chain. The cytotoxicity of the immunotoxin T101-RTA was augmented 50,000 folds in the presence of monensin [16]. In another example, an immunotoxin recognizing the neural cell adhesion molecule (SEN36-RTA) was enhanced by 12,000-fold when co-applied with monensin [37]. As a third example, the amount of 3A1-rRTA immunotoxin required to inhibit protein synthesis by 50% was decreased by 1000 folds [38]. Monensin also augmented the cytotoxic effects of a targeted toxin consisting of transferrin and saporin by 1250-fold [39], and more moderately of two gelonin-based targeted toxins directed to the gonadotropin receptor [19] and the disialoganglioside GD_2_ [32].

On the other hand, the use of monensin in vivo is hindered by poor solubility and a short half-life [40]. In order to overcome these limitations, monensin was conjugated to human serum albumin [41]. Alternatively, monensin was delivered in lipid emulsions [42], encapsulated in liposomes [43] or in poly(lactic-*co*-glycolic acid) (PLGA) polymer nanoparticles [44]. These strategies that were all conducted in conjunction with ricin A chain-based targeted toxins managed to augment the solubility and to prolong the half-life of the chemical enhancer.

Other carboxylic ionophores that have potentiated the cytotoxic effects of targeted toxins are grisorixin, lasalocid and nigericin. These three compounds enhanced the T101-RTA immunotoxin by 25,000; 33,000 and 6700 folds, respectively [16]. Significant increase in cytotoxicity was observed as well for the combination of nigericin with transferrin-RTA, anti-common acute lymphoblastic leukemia antigen (CALLA, CD10)-RTA [45] and anti-carcinoembryonic antigen (CEA)-RTA [46] targeted toxins.

### 2.3. Calcium Channel Antagonists

Another group of chemicals that were able to enhance the cytotoxic effect of targeted toxins are the calcium channel antagonists (Table 3). Verapamil was first identified as a cytotoxicity enhancer for two *Pseudomonas* exotoxin (PE)-based toxins targeted to the epidermal growth factor (EGF) receptor and the transferrin receptor [51]. Verapamil was also reported to enhance targeted toxins based on ricin A chain [52] and gelonin [19]. In order to find compounds with less in vivo toxicity, four verapamil analogs (D792; D595; D528; Sz45) were investigated. These compounds were able to enhance the cytotoxicity of targeted toxins in the range of 2−67 folds [53].

Further calcium channel antagonists that showed cytotoxicity enhancement of targeted toxins are diltiazem and methoxyverapamil, both presenting combinatorial effects with the targeted toxins HB21-PE and EGF-PE [24]. Two indolizines (SR 33287 and SR 33557) potentiated the cytotoxic effects of the anti-CD5 T101-RTA immunotoxin by 620-fold [54]. The highest enhancing effects within this group of compounds were observed in the case of perhexiline that increased the cytotoxicity of two ricin A chain immunotoxins directed against CD5 and HLA-DR class II antigens present in leukemia cells up to 2000 folds [55].

The enhancing ability of calcium channel antagonists is probably associated to the inhibition of lysosomal degradation of targeted toxins and actually does not correlate with the calcium-antagonistic activity [53]. Verapamil delays degradation in lysosomes and this could result in enhanced toxicity, alternatively it may increase the therapeutic potential of targeted toxins by some general effects on membrane permeability [51]. In the cases of perhexiline and indolizines, the delay of protein degradation may occur as a consequence of the fact that they inhibit the acid lysosomal sphingomyelinase [54]. This leads to changes in the membrane lipid composition of intracellular organelles, supposedly modulates the intracellular routing of targeted toxins and facilitates their delivery to the cytosol [55].

### 2.4. Other Organic Compounds

Other organic compounds that do not fit into the classification defined in the previous subsections have been also reported to enhance the cytotoxicity of targeted toxins. A list of these compounds is shown in Table 4. Retinoic acid enhanced the cytotoxicity of several targeted toxins comprising ricin A chain but failed to deliver diphtheria toxin and *Pseudomonas* exotoxin to the cytosol [56]. The mechanism of action of retinoic acid may be associated to the modulation of the intracellular trafficking of targeted toxins into the Golgi apparatus.

Further organic compounds have shown significant increase in the cytotoxicity of targeted toxins, however, their mechanism of action is not completely clear and different hypothesis have been formulated for each of the compounds. Brefeldin-A enhanced two ricin A chain immunotoxins and is believed to have its effect on the Golgi apparatus and on the vesicular routing [57]. The combination of *Pseudomonas* exotoxin-based immunotoxins with cyclosporin A caused remarkable synergistic cytotoxicity in several cancer cell lines, although the combinatorial effect was not linked to the immunosuppressive activity [58]. Similarly, wortmannin enhanced the cytotoxicity of saporin- and gelonin-based targeted toxins, but the effect may occur through an alternative pathway not involving the phosphatidylinositol-3-kinase inhibition [59].

Organic polymers comprise a big variety of compounds that has been investigated mainly for gene delivery [60]. Amongst this group of compounds, there is one, namely polyamidoamine, that has been considered for the enhanced cytosolic delivery of non-targeted toxins [61,62]. Further investigation of polyamidoamine effects in the context of targeted toxins would be of interest.

## 3. Enhancers of Viral and Bacterial Origin

Viruses have deployed effective intrinsic mechanisms in order to enter the cytosol of their host cells. Viruses are able to fuse directly with cellular membranes resulting in the cytosolic release of the viral particles but they are also internalized via endocytosis [65]. In case they are internalized by endocytosis, they are delivered into early or late endosomes. To exploit the cellular protein machinery, the viruses need to escape from the endosomes, a process that is mediated by specific viral fusion proteins [66] and cell-penetrating peptides (CPPs). Cell penetrating peptides, also termed protein-transduction domains, are widely used as tool to mediate the cytosolic delivery of proteins and other molecules [67,68]. One of the most prominent CPPs is the transactivating factor of the human immunodeficiency virus (HIV) [69]. There are many known different cell penetrating peptides [70]. They are frequently composed of cationic amino acids such as arginine and lysine. CPPs are widely used to augment the endosomal escape of protein therapeutics, e.g., CPPs derived from viruses have been used to deliver different proteins such as green fluorescent protein (GFP), insulin or β-galactosidase into the cytosol of cells [71,72]. As a general delivery platform, a fusion protein of the HIV transactivator of transcription (Tat) and calmodulin was designed in order to drag different calmodulin binding proteins into the cytosol of eukaryotic cells [73]. However a drawback of this approach is the non-specific delivery of protein cargo into off-target cells. Due to their small size of only several amino acids, the sequence of a CPP can be easily integrated into the sequence of targeted, recombinant anti-tumor toxins, which resulted in a targeted CPP. By inserting cleavage sites CPPs can be cleaved off after cytosolic delivery of the protein cargo. One example is the construction of a targeted anti-tumor toxin consisting of human EGF, a CCP from the hepatitis B virus and an endosomal cleavable peptide [74]. The fusion of viral CPPs to ribosome-inactivating proteins improved significantly the efficacy of these toxins against cancer cells [75,76,77]. The products are therefore optimized targeted anti-tumor toxins with fusogenic properties (Table 5).

Bacterial toxins are widely used as toxin components of targeted toxins. The most often used bacterial toxins for the design of targeted toxins are truncated versions of *Pseudomonas* exotoxin A (PE) [85]. PE comprises three domains, a receptor binding domain (Ia), a membrane translocation domain (II) and the ADP-ribosyl transferase domain (Ib and III) [86]. The truncated domain II mediates the translocation of domain Ia and III into the cytosol via the ER-associated degradation pathway [87]. The specific domains of PE were optimized by evolution in order to facilitate the efficient intracellular delivery of the ADP-ribosyl transferase domain into the cytosol [87,88]. For the design and efficient delivery of targeted toxins, it might be therefore obvious to make use of evolutionarily optimized transduction mechanisms rather than integrating artificial transduction domains into targeted toxins such as CPPs. For instance, it has been shown for diphtheria toxin from *Corynebacterium diphtheria* that the native diphtheria transduction domain (amino acids 190–390) exhibited the highest cytotoxicity in a human EGF receptor-targeted anti-tumor toxin. Fusions between several viral protein transduction domains and diphtheria toxin A chain were less cytotoxic in target cells expressing the human EGF receptor [89]. It is therefore not surprising that a large number of targeted toxins are based on *Pseudomonas* exotoxin A and diphtheria toxin [90,91,92,93,94,95,96,97,98,99,100,101]. A fusion protein of interleukin-2 and a truncated diphtheria toxin consisting of 389 amino acids called DAB(389)IL-2 was the first and to date sole targeted protein toxin that has been approved by the U.S. Food and Drug Administration under the name denileukin diftitox (Ontak^®^) [102]. A ten amino acids long motif in the transmembrane helix 1 of diphtheria toxin that is conserved in anthrax (see next paragraph) and botulinum neurotoxin serotypes mediates the delivery of the toxic domain from the lumen of early endosomes to the cytosol [103]. Using DAB(389)IL-2 it has been demonstrated that introduction of a L221E mutation into a highly conserved residue within this motif results in a nontoxic phenotype that is unable to mediate cytosolic translocation [103]. Thus, this domain acts as an effective endosomal escape enhancer.

A further intriguing approach to augment the endosomal escape process of targeted toxins is the utilization of the protective antigen of *Bacillus anthracis*. This bacterium produces a lethal toxin (anthrax toxin) that is composed of three domains: the lethal factor (LF), edema factor (EF) and protective antigen (PA). LF is a toxic zinc protease and EF an adenylate cyclase that elevates intracellular cAMP levels. PA mediates the endosomal escape by forming an acid induced pore in the endosomal membrane [104]. PA is activated by cellular furins on the cell surface. Since the PA-mediated delivery of LF and EF is a highly efficient process, attempts have been made to utilize this mechanism for targeted tumor therapies. By replacing the native furin cleavage site by tumor-specific cleavage sites, the PA-mediated endosomal escape process can be utilized for the delivery of ADP-ribosyl transferases [105,106]. The PA-mediated delivery has been also deployed for the target specific delivery of DNases such as the cytolethal distending toxin [107] into tumor cells [108]. However, although the results of these studies are promising, the strategies with modified anthrax toxins are afflicted with non-specific activation by regular cells resulting in elevated side effects.

In addition to the use of specific bacterial protein transduction domains, integral attempts have been made to co-deliver protein based cytolysins into the lysosomes of the cancer cells that have been loaded with targeted toxins. Known cytolysins are streptolysin O from *Streptococcus pyogenes* [109], perfringolysin O from *Chlostridium perfringes* [110] and listeriolysin O from *Listeria monocytogenes* [111]. In general, cytolysins are able to induce pore formation in lipid bilayers resulting in the leakage of membranes. By fusing a targeting ligand (human fibronectin domain) to listeriolysin O, a receptor-specific uptake of this construct into lysosomes was achieved [112]. This was also observed for liposomal listeriolysin O that was co-encapsulated with the type I ribosome-inactivating protein (RIP) gelonin [113]. The co-delivery of enhancers and targeted anti-tumor toxins resulted in an augmented cytosolic release of the anti-tumor toxin and increased efficacy; however, a disadvantage of this strategy is the potential immunogenicity of the enhancer. Although it is advantageous to recruit immune effector cells to the tumor site, it is a drawback when the immune response is directed against the enhancer or toxins themselves, which can, in worst case, result in anaphylactic shock.

Viral and bacterial enhancers for the endosomal escape of targeted toxins revealed great potential for the efficient cytosolic transfer. These enhancers are mostly based on particular protein domains that were optimized by evolution. As targeted toxins are protein-based therapeutics, it is straightforward to utilize viral or bacterial derived enhancer domains in recombinant fusion proteins. This is advantageous since the targeting ligand, the effector molecule (toxin) and the delivery mechanism can be integrated into a single clearly defined therapeutic compound.

## 4. Enhancers of Eukaryotic Origin

### 4.1. Proteins and Peptides

The enhancers of eukaryotic origin mainly comprise CPPs and pore forming proteins. Currently, hundreds of CPPs have been found and used in biomedical research. CPPs are derived from various proteins including transactivators of gene transcription, DNA/RNA-binding proteins, antimicrobial peptides, viral particle envelope proteins and plant circular skeletal proteins [12]. CPPs of viral origin have already been described before in this review. The most applied CPP of eukaryotic origin is penetratin, a 16-amino acid long polypeptide corresponding to the third helix of the DNA binding domain (homeodomain) of Antennapedia, a *Drosophila* transcription factor [114]. The peptide exists in its natural form, in modified forms [115] or as synthetic analogs [116]. It has been widely used for the cytosolic delivery of nucleic acids and proteins but was mainly ignored for targeted toxins [117]. Since the internalization of penetratin is receptor-independent [114] (as typical for all CPPs), it might be difficult to erase this unwanted property in targeted toxins although successfully shown for the PreS2-domain of hepatitis-B virus surface antigen [83,84]. Time will tell us whether CPPs have a future in the delivery of targeted toxins.

A completely different approach is the use of pore forming proteins. Perforins are cytolytic proteins found in the granules of cytotoxic T lymphocytes and natural killer cells that share homology with cholesterol-dependent cytolysins from Gram-positive bacteria [118]. They rather circumvent the endosomal escape instead of enhancing it. Since these proteins form pores, they cannot only be used to bring toxic proteins into the cell but are also toxic by themselves. A recombinant immunotoxin consisting of a fragment containing the N-terminal 34 amino acids of human perforin and the C-terminus of a humanized anti-cytotoxic T-lymphocyte-associated protein (CTLA-4, CD152) scFv antibody selectively depleted activated T cells to prevent transplant rejection [119].

Granzymes are potent apoptosis inducing serine proteases of cytotoxic lymphocytes. Following receptor-mediated endocytosis, perforins mediate the cytosolic uptake of granzymes. A targeted toxin consisting of vascular endothelial growth factor and mutated staphylococcal enterotoxin A was used to attract cytotoxic T lymphocytes, which then secreted perforin and granzyme B (abbreviated as GzmB, Gb or GrB) around the tumor resulting in granzyme B-mediated death of tumor cells supported by perforins [120]. Although reliant on perforins in natural attacks, granzyme B can also effectively kill tumor cells in the absence of perforin when targeted to cell surface receptors [121]. A number of research groups took advantage of this phenomenon (Table 6). Granzyme B genetically fused to H22, a humanized scFv specific for CD64, led to a target cell-specific cytotoxicity with a half-maximal inhibitory concentration (IC_50_) between 1.7 and 17 nM [122]. A similar cytotoxicity (IC_50_ between 1.2 and 6.4 nM) was achieved when using granzyme M, however, target cells were killed efficiently even in the presence of the granzyme B inhibitor serpin B9 that is often expressed in solid tumors [123]. Resistance against serpin B9 was also observed for a mutant granzyme B (R201K) [124]. The same mutant was also used with an epidermal growth factor receptor-specific scFv antibody [34]. In another immunotoxin granzyme B was fused to the humanized anti-Her2/neu scFv antibody 4D5 and for further enhancement, a fusogenic peptide (called 26) was introduced [125]. Treatment with nanomolar concentrations of GrB/4D5/26 resulted in specific cytotoxicity, induction of apoptosis, and efficient downregulation of signal pathways. Even tumor cells that are resistant to the tyrosine kinase inhibitor lapatinib or the antibody trastuzumab, and cells resistant to chemotherapeutic agents showed no cross-resistance to the GrB-based fusion proteins [125].

Ricin is a plant ribosome-inactivating protein consisting of a cell-binding B chain and a catalytic A chain. Following endocytosis, ricin is transported in vesicular carriers to the endoplasmic reticulum where the two chains are separated reductively. The A chain embeds in the membrane of the endoplasmic reticulum and then retrotranslocates across this membrane [126]. Although the B chain appears to be not involved in the cytosolic translocation, it has been shown that the presence of the B chain enhances the effect of A chain-based immunotoxins. Highly purified preparations of the two chains were separately coupled to anti-human immunoglobulin antibodies and mixtures of the resulting immunotoxins markedly synergized in vitro in their ability to kill target cells [127]. In contrast, A chain and B chain-containing immunotoxins of irrelevant specificity did not synergize with the specific immunotoxins indicating that the synergy is specific [127]. In vitro killing of target cells by either univalent antibody fragments or divalent full antibodies of rabbit anti-human immunoglobulins coupled to ricin A chain can be specifically potentiated by a piggyback treatment with ricin B chain coupled to goat anti-rabbit immunoglobulins [128]. A bispecific monoclonal antibody recognizing both CEA and RTA was tested for its ability to target the ricin A chain to CEA-expressing tumor cells alone and in combination with the B chain [129]. The antibody induced significant cytotoxicity against target cells using a ricin toxin A chain concentration below that known to be intrinsically cytotoxic. The addition of the B chain then further enhanced the cytotoxicity of the bispecific antibody [129].

### 4.2. Secondary Metabolites

In addition to proteins and peptides of eukaryotic origin, some secondary plant metabolites show a very promising potential to enhance the endosomal escape of targeted toxins. In particular some glycosylated triterpenoids (saponins) of the oleanane type isolated from *Gypsophila paniculata* L. (baby’s breath) and *Saponaria officinalis* L. (common soapwort) have the ability to specifically augment the cytotoxicity of several ribosome-inactivating proteins [131,132,133]. This augmentation is neither due to a permeabilization of the plasma membrane—it occurs at non-permeabilizing concentrations of the glycosylated triterpenoid—nor due to an increase of the rate of endocytic events, but rather due to the mediation of an enhanced endosomal escape [134,135]. Findings hinted at an interaction of the toxins with the saponins at acidic pH as it is found in late endosomes and the lysosome (pH ~5) [136]. Further, the acidic environment is even a prerequisite for the synergistic action since inhibition of vesicle acidification by bafilomycin A or chloroquine restored the survival of the tested cells [137]. It is postulated that the protonation of the glucuronic acid, which is present in this particular group of saponins, and interaction with endosomal cholesterol may play an important role [137,138]. Targeted toxins can become enhanced in their cell killing efficacy by these saponins dependent on cell line and amount of target receptor expression by 3000-fold up to 4,000,000-fold [101,139,140,141], which resulted in a broadening of the therapeutic window in mice between 10-fold and 500-fold. After injection of the targeted toxins, a substantial reduction in the tumor volume occurred and complete remissions were seen in many cases of different tumor models [142,143,144]. The tumor regression across all these studies was in average about 90% and the required dose was only 2% of the dose used for a treatment without saponins. The lower dose resulted in decreased side effects and reduced immunogenicity.

SO1861 from *Saponaria officinalis* L. and SA1641 from Saponinum album (a saponin composite from *Gypsophila* spec.) are two of the few saponins that were found to display such tremendous synergism with several type I RIPs, such as saporin [101,133], dianthin [101,145] or agrostin [132]. In contrast, the cytotoxicity of the A chain from the type II RIP ricin was only enhanced 16-fold [101] and the cytotoxicity of the bacterial *Pseudomonas* exotoxin A remained unaffected by the combinatory treatment [101,137].

## 5. Synthetic Peptide Enhancers

As described before, there are a number of peptides of viral, bacterial and eukaryotic origin that have been successfully used to enhance the cytotoxicity of targeted toxins. Additionally, peptides have been synthetically produced and evaluated for their potentiating effects.

A main group of synthetic peptides that showed significant increase in protein delivery are polyarginines. Peptides consisting of a different number of sequential arginines increased the cytosolic delivery of proteins such as green fluorescent protein by modulating protein transport in the late endosomes and lysosomes [146].

In the case of targeted toxins, a polyarginine synthetic peptide (Arg_9_) was fused to an immunotoxin containing a truncated version of *Pseudomonas* exotoxin (PE35) and directed to the carcinoembryonic antigen (CEA). The Arg_9_ peptide increased the cytotoxicity of PE35/CEA(Fv)/KDEL markedly [147]. Another synthetic peptide called pJVE doubled the cytotoxic effects of the RIP dianthin when it was delivered to cells as a fusion protein consisting of transferrin as targeting moiety, pJVE as enhancer and dianthin as toxic moiety (Tfn-pJVEDIA). The pJVE peptide interacts with the endosomal membranes and facilitates the release of targeted toxins into the cytosol [80].

## 6. Physicochemical Techniques

The endosomal entrapment of therapeutics can also be overcome by physicochemical techniques. Such techniques do not rely on the usage of molecules that modulate the intracellular trafficking of toxins by their own. Instead, they are based on the use of physical principles or chemical reactions that directly trigger the intracellular drug delivery and may disrupt the endosomal or lysosomal membranes. Some examples of these techniques are ultrasounds [148], magnetic nanoparticles [149] and plasmonic nanobubbles [150]. Thus far, most of these techniques have only been utilized for the delivery of therapeutics other than proteins such as nucleic acids or small chemotherapeutic drugs. However, a technique called photochemical internalization has been extensively studied for the delivery of targeted toxins.

Photochemical internalization is a strategy based on photodynamic therapy for the cytosolic delivery of protein therapeutics that have been trapped during their intracellular trafficking inside the endosomes and lysosomes [151,152]. The release of targeted toxins is triggered by a light-induced photochemical reaction. When targeted toxins and a photosensitizer are administered to cells, both compounds are internalized and accumulate in the endosomes and lysosomes. Then, cells are exposed to light and reactive oxygen species (mainly singlet oxygen ^1^O_2_) are produced by the photosensitizer. The chemical reactions induced by singlet oxygen lead to the disruption of endosomal membranes [153]. Subsequently, targeted toxins are released into the cytosol and they reach their intracellular target. The most common photosensitizers for photochemical internalization are disulfonated compounds based on tetrapyrrole ring systems (porphins) [10,154,155].

This approach has been proven successful in enhancing the cytotoxicity of several targeted toxins (Table 7). They were composed of either gelonin or saporin, and targeted to different cellular receptors, including CD44 [156], CD133 [157,158], chondroitin sulfate proteoglycan 4 [159], EGFR [160], epithelial cell adhesion molecule (EpCAM, EGP-2) [161,162], human epidermal growth factor receptor 2 (HER2) [163] and vascular endothelial growth factor receptor [164]. Photochemical internalization is a minimally invasive strategy for light-controlled endosomal escape of targeted toxins that can be used for many different kinds of cancer cells [10].

## 7. Discussion

Surgery, radiotherapy and conventional chemotherapy are the most important means in the treatment of cancer. While surgery is limited to accessible tumors and in most cases is not suitable for metastasized cancer, radiotherapy and chemotherapy are accompanied by undesired side effects and adequate efficacy is often missing due to formation of resistances. In the last decades targeted tumor therapies that address more specific properties of tumor cells were developed; they mainly comprise receptor tyrosine kinase inhibitors [169] and antibody-based therapies [2]. The cytotoxic effect of antibodies can be mediated by direct effects of the antibody such as acting as receptor antagonist or by inducing apoptosis, by indirect effects such as complement dependent cytotoxicity, antibody-dependent cell-mediated cytotoxicity or modulation of T cell functions, and by specific effects on the tumor environment in particular on vascularization [170]. To augment the effect of antibodies, they were linked to cytotoxic substances, either small molecules or protein-based toxins [171]. The antibodies can be replaced by other targeting ligands such as cytokines or growth factors, which also results in targeted toxins [171]. Conjugates with small toxic molecules are more advanced than immunoconjugates with protein-based toxins, but they still reveal problems with linker chemistry, conjugate homogeneity and overall stability [172,173,174]. Moreover, the toxic substances in antibody small drug conjugates are typically competitive inhibitors, whereas the protein toxins are enzymes. Therefore, only a small number of these toxic proteins must enter the cytosol to mediate cell death [175]. Although protein-based targeted toxins appear to have unbeatable advantages including high target-specific toxicity and recombinant production of homogenous molecules, no antibody-targeted protein toxin for tumor therapeutic applications has been launched to date and only one ligand-targeted fusion protein, denileukin diftitox, was approved by the U.S. Food and Drug Administration [102]. As described in this review article, the low cytosolic uptake of the toxin components is a major concern regarding therapy with targeted toxins. Reasons for this are poor internalization, recycling to the cell surface and lysosomal degradation [6,7,8]. Thus, the advantage of the high efficacy of the protein toxin is undermined by the necessity to apply high doses of the toxin to gain the desired rate of cytosolic translocation. It is notable that denileukin diftitox that uses its evolutionarily developed intrinsic endosomal escaper enhancer did not lead to a breakthrough in the application of targeted toxins even 17 years after approval.

**Figure 1:**
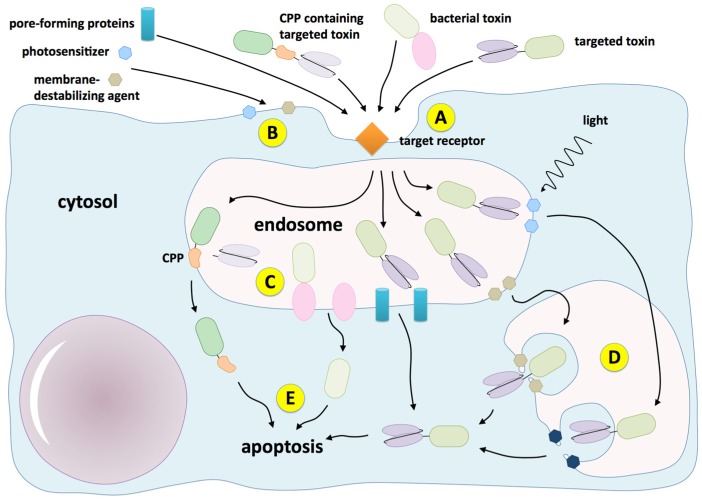
Endosomal escape enhancers for targeted toxins: (**A**) as native bacterial toxins, targeted toxins also bind to the target receptor and are internalized; (**B**) membrane-destabilizing agents and photosensitizers typically enter cells by passive diffusion and accumulate preferably in the endosomal membrane; (**C**) cell-penetrating peptides (CPPs), native bacterial translocation domains and pore-forming proteins directly mediate the entry of the toxins into the cytosol; (**D**) membrane-destabilizing agents and photosensitizers rather destroy the membrane integrity resulting in leaky endosomes, which allows translocation of the toxins; and (**E**) the toxins affect vital metabolic pathways in the cytosol finally resulting in apoptosis of the cell.

It is therefore obvious that efficacy enhancers are important tools to improve the endosomal escape rate and accordingly decrease the dosage. All the efficacy enhancers presented here (Figure 1), independent of their origin and structure, show substantial enhancer effects, however, none of them is tumor target specific by itself; they all depend on the specificity of the targeted toxin. In the case of off-target effects of the toxin, these unwanted effects might also be enhanced and result in increased side effects. This can be the case due to lack of antibody specificity resulting from the presence of the target receptor on healthy tissue (target dependent toxicity) or due to targeted toxin binding to other cell surface components rather than specifically to its target receptor (target independent toxicity) [176]. Therefore, additional measures are required to prevent enhancement of off-target effects.

Another problem with enhancer substances concerns pharmacokinetic issues. In most cases, the enhancer substance is applied independently from the drug and has its own behavior in liberation, absorption, distribution, metabolization and excretion. Since both compounds, the enhancer and the targeted toxin, must be at the site of interaction at the same time, a careful synchronization of both kinetics is required. Moreover, chemical enhancers, secondary metabolites and photosensitizers are non-specifically taken up by off-target cells. All these facets make it very difficult to determine the mutual dose dependency of both substances, the spatial and temporal distance of both applications, and the therapeutic window.

There are mainly two solutions for the mentioned problems, first, to also target the enhancer molecules to the tumor cells, but independently of the targeted toxin, and second, to combine enhancer and targeted toxin in a single drug molecule. Neither solution can solve both problems discussed before. The first solution avoids off-target uptake of the enhancer and therefore minimizes undesired enhancement of misguided targeted toxins to regular cells but cannot ensure synchronization while the second solution results in synchronization of the kinetics but will still enhance the cytotoxic effect if the antibody binds and is internalized by an off-target cell.

Cell penetrating peptides can be fused to the toxin moiety to make them target cell specific [80,83,84]. The idea is to inactivate the penetrating property until the antibody has been bound to the target receptor and internalized. A possible solution is to place the CPP between targeting moiety and toxin and include an endosomal cleavable peptide that leads to the release of the targeting moiety inside the endosomes, which in turn results in exhibition of the CPP [83,84]. Chemical enhancers and secondary metabolites can be encapsulated into targeted nanocarriers; a pH-sensitive endosomal stimulus can then result in controlled release [177,178]. The nanocarriers can be in addition spiked with the targeted toxins or applied independently, each variant associated with the pros and cons discussed before. Further specificity might be achieved by use or design of toxins that are predominantly activated only in tumor cells as described for apoptin [179,180]. In the case of photochemical internalization, nothing happens even in the case of colocalization of drug and enhancer in off-target cells. Light exposure then results in disruption of the endosomal membrane. Additional specificity is thus being obtained by only illuminating the site of the tumor [151]. This is however achieved at the expense of completeness in the treatment of metastases since unidentified micrometastases cannot be embraced by this system except for unenhanced targeting. This problem might be solved by targeted bioluminescence, however, firefly luciferase bioluminescence does not generate sufficient photons to induce photodynamic toxicity [181].

The use of enhancers leads to a further general problem, the immunogenicity of drugs. In particular, peptide- and protein-based enhancers can contain immunogenic epitopes, and small molecule enhancers, even if not immunogenic by themselves, might boost a possible immune response against the targeted toxin. Strategies to deimmunize enhancers might be applied in the same way as described for targeted toxins [96]. Wherever possible, human protein sequences should be used as exemplified by humanized antibodies or human toxins such as angiogenin or granzymes.

## 8. Conclusions

Antibody targeted delivery of protein toxins to tumor cells is one of the most promising ideas in the fight against cancer, however, the fact that decades of research have not resulted in any approved antibody-targeted protein toxin for tumor therapy shows that cytosolic protein delivery is a formidable challenge. The main barrier is the endosomal membrane that prevents release of the toxin before degradation or recycling. To overcome this obstacle, a number of strategies were developed that can be summarized as endosomal escape enhancers. The nature of these enhancers is multifarious including chemical substances, cell-penetrating peptides, protein domains, secondary metabolites, and light-sensitive substances. Until now, it is not finally solved how spatio-temporal synchronization of enhancer and drug should be accomplished, and how enhancement of drugs that were misdirected into regular cells should be prevented. Further ideas are definitely required to solve the conflict between linked enhancer/drug delivery and independently targeted enhancers. This might include new controlled delivery techniques and novel tumor activated toxins.

## Figures and Tables

**Table 1:** Ammonium chloride and lysosomotropic amines that enhance the cytotoxicity of targeted toxins. All listed substances act by increasing the pH in the lysosomes and thus preventing degradation.

Enhancer	Toxin	Antigenic Target	Targeted Toxin	Enhancer Concentration	Max. Enhancement Factor	Ref.
Amantadine	ricin A chain	CD5	T101-RTA	1 mM	1180	[16]
	saporin	CD5	OKT1-SAP	1 mM	4	[25]
		CD5	T101-SAP	1 mM	4	[25]
		CD5	SOT1a-SAP	1 mM	4	[26]
Ammonium chloride	ricin A chain	CD5	T101-RTA	10 mM	6700	[16]
		Thy 1.2 (CD 90)	AT15E-RTA	10 mM	6	[16]
		melanotransferrin	96.5-RTA	10 mM	42	[16]
		CD7	WT1-RTA	6 mM	100	[27]
		CD25	anti-TAC-RTA	10 mM	100	[28]
		cCLLa	CLL_2m_/RTA	10 mM	80	[29]
		EGFR	EGF-RTA	10 mM	12	[30]
		CD3	WT32-RTA	6 mM	Significant increase	[31]
Chloroquine	gelonin	LH receptor	lutropin-gelonin	9.6–29 µM	15	[19]
		GD_2_	gelonin-14G2a	10 µM	10	[32]
	pokeweed antiviral protein	transferrin receptor	5E9-11-PAP	10–100 µM	65	[20]
		T cells	T3-3A1-PAP	10–100 µM	65	[20]
		CD19	B43-PAP	40 µM	Significant increase	[33]
	ricin A chain	CD5	T101-RTA	100 µM	2500	[16]
	saporin	transferrin receptor	saporin-transferrin	10 µM	Significant increase	[21]
	granzyme B	EGFR	Gb(R201K)-scFv1711	50 µM	3	[34]
Dimethylamine	ricin A chain	CD5	T101-RTA	10 mM	3300	[16]
Lipopolyamines	saporin	urokinase receptor	uPA-saporin	5 µg/mL	83	[23]
Methylamine	ricin A chain	CD5	T101-RTA	10 mM	13,300	[16]
Quinacrine	gelonin	LH receptor	lutropin-gelonin	2.6–7.6 µM	15	[19]
Trimethylamine	ricin A chain	CD5	T101-RTA	10 mM	80	[16]
β-Glycylphenyl-naphthylamide (GPN)	*Pseudomonas* exotoxin	transferrin receptor	HB21-PE	10–20 µg/mL	9	[24]
		EGFR	EGF-PE	10–20 µg/mL	6	[24]

**Table 2:** Carboxylic ionophores that enhance the cytosolic uptake of targeted toxins. The compounds in the list presumably take effect by augmenting the pH in the lysosomes and blocking the lysosomal degradation of targeted toxins.

Enhancer	Toxin	Antigenic Target	Targeted Toxin	Enhancer Concentration	Max. Enhancement Factor	Ref.
Grisorixin	ricin A chain	CD5	T101-RTA	50 nM	25,000	[16]
Lasalocid	ricin A chain	CD5	T101-RTA	1 µM	33,000	[16]
Monensin	gelonin	LH receptor	lutropin-gelonin	0.3–2.9 µM	15	[19]
		GD_2_	gelonin-14G2a	100 nM	10	[32]
	ricin A chain	CD5	T101-RTA	50 nM	50,000	[16]
		Thy 1.2	AT15E-RTA	50 nM	4	[16]
		p97	96.5-RTA	50 nM	420	[16]
		transferrin receptor	Tfn-RTA	10–100 nM	30,000	[45]
		CD10	anti-CALLA-RTA	10–100 nM	Significant increase	[45]
		CD25	anti-TAC-RTA	25 nM	400	[28]
		CEA	anti-CEA-RTA	0.5–1 µM	Significant increase	[46]
		p55	260F9-rRTA	100 nM	34	[47]
		gp74	45-2D9-RTA	0.5 µM	Significant increase	[48]
		p55	260F9-rRTA	10–100 nM	Significant increase	[38]
		CD7	3A1-rRTA	10–100 nM	Significant increase	[38]
		transferrin receptor	R17-217-rRTA	10–100 nM	Significant increase	[38]
		transferrin receptor	Tfn-RTA	900 nM	500	[39]
		transferrin receptor	OKT9-RTA	900 nM	3300	[39]
		transferrin receptor	OX26-RTA	900 nM	330	[39]
		N-CAM	SEN36-RTA	100 nM	12,000	[37]
		MUC1	BrE-3-RTA	100 nM	100	[49]
		transferrin receptor	454A12MAb-RTA	10–100 nM	4	[50]
	saporin	transferrin receptor	Tfn-So6	900 nM	1250	[39]
Nigericin	ricin A chain	CD5	T101-RTA	10 nM	6700	[16]
		transferrin receptor	Tfn-RTA	10–100 nM	Significant increase	[45]
		CD10	anti-CALLA-RTA	10–100 nM	Significant increase	[45]
		CEA	anti-CEA-RTA	0.5 µM	Significant increase	[46]

**Table 3:** Calcium channel antagonists enhancing the cytotoxicity of targeted toxins. These substances probably cause their effect by blocking the lysosomal degradation of targeted toxins and modulating their intracellular trafficking.

Enhancer	Toxin	Antigenic Target	Targeted Toxin	Enhancer Concentration	Max. Enhancement Factor	Ref.
Diltiazem	*Pseudomonas* exotoxin	transferrin receptor	HB21-PE	10–20 µg/mL	6	[24]
		EGFR	EGF-PE	10–20 µg/mL	8	[24]
Indolizine (SR 33287; SR33557)	ricin A chain	CD5	T101-RTA	5 µM	620	[54]
		Thy 1.2	AT15E-RTA	5 µM	84	[54]
Methoxyverapamil (D-600)	*Pseudomonas* exotoxin	transferrin receptor	HB21-PE	5–20 µg/mL	12	[24]
		EGFR	EGF-PE	5–20 µg/mL	20	[24]
Perhexiline	ricin A chain	CD5	T101-RTA	1–5 µM	2000	[55]
		HLA-DR class II	HNC-241-RTA	1–10 µM	100	[55]
Verapamil	gelonin	LH receptor	lutropin-gelonin	10–41 µM	15	[19]
	*Pseudomonas* exotoxin	transferrin receptor	HB21-PE	2.5–20 µg/mL	11	[24]
		EGFR	EGF-PE	10–20 µg/mL	40	[24]
	ricin A chain	transferrin receptor	454A12-rRTA	20 µg/mL	25	[52]
		p55	260F9-rRTA	20 µg/mL	8	[52]
		HER2	454C11-RTA	20 µg/mL	Significant increase	[52]
		cCLLa	CLL_2m_/RTA	20 µg/mL	80	[29]
Verapamil analogs (D792; D595; D528; Sz45)	*Pseudomonas* exotoxin	transferrin receptor	HB21-PE	20 µM	35	[53]
	ricin A chain	transferrin receptor	454A12-rRTA	1–20 µg/mL	67	[53]
		p55	260F9-rRTA	20 µM	Significant increase	[53]

**Table 4:** Other organic compounds that enhance the cytotoxicity of targeted toxins. Listed substances induce their effect on different intracellular vesicular compartments. For some of the compounds, the exact mechanism of action has not been completely elucidated yet.

Enhancer	Toxin	Antigenic Target	Targeted Toxin	Enhancer Concentration	Max. Enhancement Factor	Ref.
Brefeldin-A	ricin A chain	transferrin receptor	454A12-rRTA	0.05–0.5 µg/mL	Significant increase	[57]
		p55	260F9-rRTA	0.025–0.05 µg/mL	Significant increase	[57]
Cyclosporin A	*Pseudomonas* exotoxin	EGFR	425.3PE	2 µM	Significant increase	[58]
		MUC1	BM7PE	2–4 µM	40	[58]
		EpCAM (EGP-2)	MOC31PE	2 µM	Significant increase	[58]
	ricin A chain	gp70	2F10-RTA	25 mg/kg (in vivo)	100	[63]
		CD5	T101-RTA	4 µmol/mL	101	[64]
Cyclosporin SDZ PSC 833	ricin A chain	CD5	T101-RTA	4 µmol/mL	105	[64]
Retinoic acid	ricin A chain	transferrin receptor	454A12-rRTA	10 µM	10,000	[56]
		transferrin receptor	Tfn-rRTA	10 µM	1000	[56]
		B cells	M6-rRTA	10 µM	Significant increase	[56]
		p55	260F9-rRTA	10 µM	Significant increase	[56]
Wortmannin	gelonin	bFGFR	bFGF-gelonin	1–10 µM	Significant increase	[59]
	saporin	bFGFR	bFGF-SAP	1–10 µM	Significant increase	[59]
		EGFR	HBEGF-SAP	1–10 µM	Significant increase	[59]
		bFGFR	11A8-SAP	1–10 µM	Significant increase	[59]

**Table 5:** Viruses and virus peptides showing enhancement effects on the cytotoxicity of targeted toxins. It is expected that the mechanism is similar to the natural process of fusogenic peptides of viral origin when viruses enter the cytosol of infected cells.

Enhancer	Application	Toxin	Antigenic Target	Targeted Toxin	Enhancer Concentration	Max. Enhancement Factor	Ref.
Adenovirus	whole virus	*Pseudomonas* exotoxin	EGFR	PE-EGF	2 × 10^9^ pfu/mL	10,000	[78]
		ricin A chain	CEA	anti-CEA-RTA	3 × 10^8^ pfu/mL	33	[46]
Penton base protein (adenovirus capsid protein)	whole virus	*Pseudomonas* exotoxin	EGFR	PE-EGF	9 × 10^3^ viruses/cell	Significant increase	[79]
KFT25 (N-terminus of Protein G)	viral peptides (fusion proteins)	dianthin	transferrin receptor	Tfn-KFT25-DIA	≤30,000 ng/mL	3.8	[80]
		ricin A chain	transferrin receptor	Tfn-KFT25-RTA	≤10 pM	20	[81]
HA23	viral peptides (conjugates and free peptides)	ricin A chain	gp120	anti-gp120(HIV)-RTA-HA23	0–300 µg/ mL	5	[82]
PreS2-domain of hepatitis-B virus surface antigen (TLM)	viral peptides (fusion protein)	saporin	EGFR	saporin-TLM-EGF	≤100 nM	1 (in vitro) 2.2 (in vivo)	[74,83]
		angiogenin	CD64	anti-CD64-TLM-angiogenin	≤100 nM	20	[84]

**Table 6:** Toxic proteins of eukaryotic origin that have been used in targeted toxins. The pore forming proteins oligomerize in a Ca^2+^ dependent manner to form pores on the target cell. They in principle fulfill both functions, the toxic effect and the ability to overcome the membrane barrier, in one molecule. Granzymes are apoptosis inducing serine proteases that are supported by pore forming proteins to enter the cells. An enhancement factor cannot be provided here since efficacy and enhancement are inextricably linked with each other in these systems.

Enhancer = Toxin	Antigenic Target	Targeted Toxin	Enhancer = Toxin Concentration (IC_50_)	Ref.
Perforin (N-terminal 34 amino acids)	CTLA-4 (CD152)	hS83P34	200–1000 nM	[119]
Perforin / Granzyme B	VEGF	VEGF-SEA (D227A mutant)	released by attracted immune cells	[120]
Granzyme B	Lewis Y	GzmB-dsFv-B3	35–140 nM	[121]
	CD64	Gb-H22(scFv)	1.7–17 nM	[122]
		Gb(R201K)-H22(scFv)	4–7 nM	[124]
	CD30	Gb(R201K)-Ki4(scFv)	1.7 nM	[130]
	EGFR	Gb(R201K)-scFv1711	133 nM	[34]
	HER2	GrB/4D5	242–629 nM	[125]
		GrB/4D5/26	29–93 nM	[125]
Granzyme M	CD64	Gm-H22(scFv)	1.2–6.4 nM	[123]

**Table 7:** Targeted toxins enhanced by photochemical internalization. The photochemical reaction of a photosensitizer triggered by light exposure causes the disruption of endosomal membranes. This results in the release of targeted toxins into the cytosol and increases their cytotoxicity.

Photosensitizer (Enhancer)	Toxin	Antigenic Target	Targeted Toxin	Photosensitizer (Enhancer) Concentration	Ref.
AlPcS_2a_	gelonin	CSPG4	scFvMEL/rGel	5 µg/mL	[159]
TPCS_2a_	gelonin	EGFR	rGel/EGF	0.1–0.4 µg/mL	[160]
		HER2	MH3-B1/rGel	0.1–0.4 µg/mL	[163,165]
		VEGFR	VEGF121/rGel	0.4 µg/mL	[164]
	saporin	CD133	CD133/1 (AC133)-saporin	0.4–1 µg/mL	[157,158]
		CD133	CD133/2 (293C)-saporin	0.2–1 µg/mL	[157]
		CD44	IM7-saporin	0.35–1 µg/mL	[156]
		EpCAM (EGP-2)	3–17I-saporin	0.35 µg/mL	[162]
		HER2	Trastuzumab-saporin	0.2 µg/mL	[166]
TPPS_2a_	gelonin	EpCAM (EGP-2)	MOC31-gelonin	0.3–1 µg/mL	[161]
	saporin	EGFR	Cetuximab-saporin	0.1–1 µg/mL	[167]
		EGFR	EGF-saporin	0.1–0.2 µg/mL	[168]
